# Inter-embodied parental vigilance; the case of child food allergy

**DOI:** 10.3389/fsoc.2023.1213769

**Published:** 2023-07-27

**Authors:** Marie-Louise Stjerna, Geraldine Brady

**Affiliations:** ^1^Department of Special Education, Faculty of Social Sciences, Stockholm University, Stockholm, Sweden; ^2^Department of Social Work, Care and Community, School of Social Sciences, Nottingham Trent University, Nottingham, United Kingdom

**Keywords:** inter-embodiment, child health, food allergy, parental perspective, parental vigilance

## Abstract

There is developing interest in issues of embodiment in studies of children, health and illness. We take our point of departure in the parent-child-health/illness triad to explore the embodied aspects of parental vigilance in parenting children who have a food allergy, utilizing the concept of inter-embodiment. Drawing on a focus group study with parents in Sweden the analysis reveals that this vigilance can be seen as the embodied manifestation of concern for children's bodies in perpetual liminality, when constantly exposed to allergens and the risk of becoming ill. We argue that the lens of inter-embodiment, with a focus on bodies in relation, captures how parents lived experience of managing food allergy intertwines with that of their children in the parent-child-health/illness triad. The analysis uncovers a form of embodied knowledge that is often not verbalized, offering potential for new understandings of parent-child relations that center on chronic child health conditions.

## Introduction

In contemporary society, parents' understandings and ways of addressing child health are influenced by “warnings and anxieties about risks and dangers to which children may be susceptible” (Lupton, 2012, p. 45), and parents are found to be highly aware of their responsibility for managing their children's health (Lee et al., 2010; Lupton, 2011). As parents are primarily the mediators of children's health and well-being, the parent-child relationship can be depicted as a triad: parent-child-health/illness. Characteristic of this triad is that any imperative to observe and monitor the child's body and behavior becomes intensified in cases of child illness. Studies indicate that in addition to a “normal” watchfulness, parents develop a “parallel vigilance” looking for signs of illness, as is indicated in cases of child Type 1 diabetes (Niedel et al., 2013). Parents seem to base their understandings of their children's health on detailed observations of the child's body. They explore threats to the child's health, act upon those threats and evaluate how their actions influence their children's health (Olin Lauritzen, 1997; Lupton, 2011). Also, parents separate what they regard as “natural” and “cultural” in infants' bodies, such as childrens' diseases contra medical interventions. These understandings of the body as a dynamic system, where the inner body is connected to the outside world in complex ways, further enhance the parental responsibility. As it is difficult to comprehend and predict bodily consequences of actions related to children, today parents seem to be “responsible for everything” but at the same time “powerless” (Brownlie and Leith, 2011, p. 206). Parenting a child who is experiencing illness can amount to “living” the child's illness, as the lives of parents and children are intertwined in various ways in terms of caring, responsibility and, not least, bodily intimacy. Earlier, the first author and colleagues analyzed social aspects of the phenomena of parental vigilance in studies with parents of children with food allergy in a range of everyday contexts as well as cross-culturally (Stjerna et al., 2014, 2017). In this paper we are pursuing further understandings of the lived experience of parenting children who have a food allergy, through an exploration of the more embodied aspects of this parental vigilance, by utilizing the concept of inter-embodiment.

### The case of child food allergy

The food allergy condition offers a productive case to explore parental vigilance and bodily aspects within the parent-child relationship as food allergy is characterized by a constant parental responsibility to prevent allergic reactions, together with uncertainties involved in the diagnosis and management of the condition. Also, since reactions toward food allergens produce visible bodily manifestations such as rashes, swollen lips, shortness of breath and cough, food allergy is advantageous to study from an embodiment perspective. Further, the concept of inter-embodiment is useful to explore parental vigilance in cases of child illness since this concept focuses on bodies in relation (see e.g., Lupton, 2012). Food allergy is of increasing public health concern and affects up to 12 percent of the child population in the Western world (Burks et al., 2012), and is likely to increase globally in the coming decade (Prescott et al., 2013). It is well known that parents of food allergic children experience anxiety and fear due to the constant vigilance needed to prevent allergic reactions (Gillespie et al., 2007). The only treatment is avoidance of certain foods, as well as management of symptoms. The most common food allergies are to milk-protein, egg and nuts, but almost any food can cause allergic reactions (Arias et al., 2009). Allergic reactions to food typically occur shortly after exposure, with symptoms varying from mild oral itch to life-threatening anaphylaxis (a potentially fatal reaction involving multi-organ systems). Children with risk of severe allergic reactions are therefore given adrenaline auto-injectors to be carried all of the time (Simons, 2010). Even when given a food allergy diagnosis, the condition involves uncertainties. It is possible to determine if an individual reacts to an allergen with a blood- or skinprick test or an oral food challenge, but none of these tests can predict how severe a reaction could be (Hu et al., 2008).

### The lived body and the concept of inter-embodiment

The lived body, although individuated and autonomous, in contrast to an “abstract” medical body (see e.g., Armstrong, 1983) actually experiences the intertwining with other bodies. Here we draw on Merleau-Ponty's ((2002[1962])) understanding of perception and the lived body. According to Merleau-Ponty we experience ourselves, others and the world *through* our bodies. The intentional body not only responds to the world but creates meaning about different phenomena and the world through its sensations. Following the intercorporeality of Merleau-Ponty ((2002[1962])) the body is at once both subject and object; entangled and interconnected to other bodies. Springgay (2005) states that by taking the point of departure in Merleau-Ponty's ontology “a re-conceptualization of body knowledge must consider the possibilities of interactions *between* bodies—knowledge as intercorporeality” (page 37). Lupton, drawing from the work of Merleau-Ponty, uses the term inter-embodiment as a “concept of relation” that highlights the ways bodies “live alongside and in response to others” bodies' (Lupton, 2012, p. 39). During infancy, and also later in the child's life, this inter-embodiment is experienced through embodied caring practices such as feeding- or hygiene practices carried out as part of everyday parenting (Lupton, 2012). However, as has been pointed out within the sociology of childhood (James et al., 1998), children are not just recipients of care or passive bodies that parents “act upon”, but active social agents who also shape their bodies and social lives (Mayall, 2002; Christensen and James, 2008; Brady et al., 2015). Thus, the parent-child-health/illness triad can be seen as characterized by a dynamic relation between active parents *and* children. It is also depending on the health issues at stake as well as changing relationships over time as the child grows older and their ability to exercise agency increases in the context of intra-generational dependencies (Brady et al., 2015; Mayall, 2015; Holloway et al., 2019). Children's bodies need to be understood through the practices, materials and processes that produce and maintain them. Indeed, bodily relations, which emerge within power-inequalities, are central to the understanding of inter-generational relations (Mayall, 2015; Holloway et al., 2019). Today there is growing interest in children and embodiment within the field of childhood studies and children's geographies (Prout, 2000; Colls and Hörschelmann, 2009; Lupton, 2012) and studies have shown that aspects of embodiment and inter-embodiment within the parent child relation can surface in fruitful ways in interviews with parents about their children's health (Olin Lauritzen, 1997; Lupton, 2011; Henderson et al., 2020).

### Aim

It is against this backdrop that in this paper we will address issues of inter-embodiment in the parent-child-health/illness triad, drawing on the case of child food allergy. More specifically the aim is to explore the character of the inter-embodied parental vigilance in parenting children with food allergy; how this is reflected in parents accounts about their lived experience of managing their child's illness within the parent-child-health/illness triad.

## A focus group study of parents of children with food allergy

This paper draws on a study that was carried out in 2009–2010 in Sweden and included ten focus group interviews with 31 parents (25 mothers and six fathers) of children with food allergy, aged 1–17 years (Stjerna et al., 2014). The study was approved by the Ethics Committee at the Karolinska Institutet (Nr: 2008/569-31, 2012/1051/32)^1^. All children had been diagnosed with a food allergy, either single food or multiple, varying from mild allergy to potentially life-threatening allergy, and most were prescribed an adrenaline auto-injector to be administered in case of severe reactions. Focus group interviews are particularly suited to explore shared knowledge, opinions and underlying attitudes with a group of people who share similar experiences and/or characteristics. The interactions which take place in focus groups are in many ways similar to everyday interaction in more informal settings. For example, speech acts are mostly spontaneous, overlapping speech occurs and a good story is often used as an example of something. One of the advantages of the focus group method is that the group dynamics in the interview situation can stimulate reflection as participants are allowed to build on each other's experiences and thoughts as they are expressed in the on-going conversations (Marková et al., 2007). The focus group interviews took place at the two hospitals where the children were patients and were moderated by the first author. A physician participated as a silent observer and answered medical questions at the end of each session, important for ethical reasons. The sessions lasted for 1.5–2 h and were digitally recorded with the parents' permission. The parents were asked to share their experiences of their lives with a food allergic child and a topic guide was used, covering everyday management of the allergy at home, nursery, school and other arenas. The parents expressed that they appreciated the opportunity to share experiences in a group with other parents of children with food allergy and that they largely lacked such opportunities in their daily lives. They presented detailed accounts of their experiences, often as a course of events resulting in sequences with a narrative character where the individual parent “takes the floor”, sometimes in an alliance with another parent/s who fills in or asks a question. There are also sequences when several participants, in a collective way, jointly contribute to a topic. All parents took part in the conversation, albeit a few contributed less to the conversation than others. This can be seen as typical of focus group interviews, but here, these parents referred to differences in the severity of their child's food allergy; stating that the child's food allergy was not such a big problem in the family and that other parents have more to contribute. Throughout the focus group sessions though, parents positioned themselves as “we” in contrast to “others”, who did not share their experiences of managing child food allergy.

### Analysis

Since this study was conducted in 2009–2010 there is evidence that food allergy is increasing in Western countries; the “second wave” started in the in the early 2000s and is also likely to spread globally in the coming decade. This development will have a major influence on healthcare provision of specialist allergy services worldwide (Prescott and Allen, 2011; Prescott et al., 2013). There is also concern that allergies that tended to outgrow in earlier generations of children, such as egg and milk allergies, are less likely to outgrow among children of this second wave (Prescott and Allen, 2011). Thus, food allergy continues to be of increasing public health concern. Since there is no cure those who have the allergy have to manage it on a daily basis in a number of situations, and there is no reason to suspect that parents of children with food allergy or children's experiences of living with food allergy have changed in fundamental ways since this study was conducted. To our knowledge there are a dearth of studies that shed light on inter-embodiment aspects from a parental perspective in cases of chronic child illness and this study offers empirical material to explore such experiences. A first analysis of this material showed that bodily aspects were salient in parents' talk. This urged for a deeper analysis of the meaning of these bodily aspects as a part of the parental vigilance.

This re-analysis is based on the already transcribed and anonymised material, and no other information about the individual participants was used by the researchers. The analysis was carried out by the first author, who had collected the data and thus had a first-hand inside perspective on the material, in collaboration with the second author. Although, the interaction in the focus groups was not the specific focus of this analysis, attention was also paid to how the interaction in the groups produced the data (Morgan, 2010). The analysis entailed that topical episodes were identified in the material; sequences that were held together internally by the content during a sequence of time (Marková et al., 2007). These episodes are chosen as they revolve around bodily aspects of the parental vigilance when parenting children with food allergy. In a second step these episodes were brought together into more overarching themes. From the point of departure of an embodiment perspective and utilizing the concept inter-embodiment the authors continuously discussed the emergent themes. Finally, three major themes that were of relevance to the issue of inter-embodied parental vigilance were identified. These are;

1. How parents identify and understand signs of food allergy by observing the child's body and how inter-embodiment aspects are part of those identification processes
2. How parents understand allergy risk as localized to the child's body, as something internal, and how they make sense of these inner, invisible aspects
3. How parents support their children to identify and interpret food allergy signs and respond to bodily reactions.

## Results

Generally speaking, the infant's body is characterized by its' vulnerability and being “at risk” and inter-embodiment is part of parents' care-taking practices (Olin Lauritzen, 1997; Lupton, 2012). However, with the case of illness in children, parental surveillance entails responses which are related to the ways the particular illness manifests in everyday life. The food allergy case demonstrates how the parents pay attention to deviances from the child's normal functioning and behavior; the body exterior.

### To read the body of the child

The parents use their own senses to identify signs of illness. They describe how they *look* at the child's skin, face, body movements as well as behavior—*listen* to the child's breathing or wheezing or coughing. There is the awareness that allergic reactions potentially are life-threatening:

it has affected her breathing, you can hear how it “gurgles”, it sounds difficult and it is difficult for her to breath, but she has always had enough oxygen in her blood so luckily she has never experienced this as very uncomfortable (6 years old, allergic to egg, hazelnuts, cashew, pistachio)

This mother points out that when it comes to a reaction that affects breathing, the oxygen level in the blood is crucial. This indicates there is a moment when the reaction might get worse, pointing to the unpredictability of a reaction. Further examples also demonstrate how the child's experiences of having severe reactions might influence the child's embodied agency; both during the course of events and afterwards. Some of the parents describe that their children express stress, fear and bodily resist when entering the ambulance or being treated in emergency care. One father said that when his daughter had difficulties breathing he gave her adrenaline and called for the ambulance. His daughter, 4 years old, who had experienced a number of medical examinations, first refused to go into the ambulance: “she was holding so tight to me that I could hardly breath, so much strength in this little body”. Some of the parents also describe how they witness dramatic changes in their children in cases of severe reactions. This mother depicts severe reactions in her daughter: she can *see* how the rashes grow all over her body, she can *hear* a “strange cough”, she can *observe* how her behavior changes:

she gets so aggressive, she can hardly breath, because her chest gets tight, she still manages to wrestle her way out, even though there are fifteen people in the emergency room, but after a while she comes around. Molly^2^ “turns up” and one of the nurses says, hello, there you are, they are so used to this, but I just wonder where she went (9 years old, allergic to cereals, shellfish and nuts)

The above excerpt shows that a severe reaction can be compared to a sudden metamorphosis of the child. This mother says that it is “so difficult” to see her daughter in these life-threatening situations; she can “just hope that it goes well”. Adopting the concept of inter-embodiment to episodes of emergency care could be understood such as parents experience limited control over their child's body and life during those episodes. Parents can indeed give their children adrenaline and call for help but then they have to put their trust in others. The mother in the above example says that after such difficult incidents she has noticed that her daughter is worried and reluctant to eat. Another example of the way that the allergy can influence the child's relation to food is from another mother who explains that her daughter had a reaction when she had a lasagne and now links this dish with her experiences of emergency care:

she usually loves lasagne, but when she says I am not hungry, I know immediately we will have something else, she becomes very scared because she has had two bad experiences at the emergency department (10 years old, allergic to milk-protein and egg)

Some parents describe that their children develop a more general aversion to food and in some situations refuse to eat, which could be understood as a strong bodily response to difficult illness experiences. This mother says that her son has had so many bad experiences that he has developed an aversion against eating: “he weighs fifteen kilos, he is really small and he cannot eat and he does not want to eat because food is associated with something that hurts him”. He will soon be 5 years old and only recently have the family been remitted to a dietician, she adds. Further, to learn to recognize how their child's condition presents and to recognize these signs of illness is knowledge that the parents develop over time:

it was actually at nursery (first allergic reaction) when he was given the wrong type of pasta, with wheat, but did not react until an hour later, and I thought he would react at once, but now I have learned (8 years old, allergic to milk-protein, wheat, barley rye, oatmeal, egg, hazelnut, peanut)

But even if the typical pattern is learnt, there is always uncertainty regarding how severe the reaction will be at this particular time, something completely “new” can happen. In spite of a diagnosis, parents express an uncertainty regarding their child's condition; stating “this is what we know right now”. This implies that it is possible to discover new allergens and receive a revised diagnosis. Furthermore, the allergy could affect closeness between the child and others since it requires such a small amount of an allergen to have a reaction and some children may react on skin contact. In this way the boundaries between the child and others bodies in some situations need to be maintained and observed. Here is an example of a mother's recollection of when she first discovered her son's allergy. A touch from a sibling during family cooking is enough to trigger a reaction:

the first time was when we made pancakes, and his older brother participated in mixing all ingredients, and then went up to Martin who was sitting in his baby chair and patted him, Martin didn't eat anything himself, he was just patted, but that was enough to trigger a reaction, and then we went to the emergency department (2 years old, allergic to egg)

Parents also have to consider the social risks that their children encounter due to their allergy, foremost in settings outside home. One mother says that her son (9 years old, allergic to milk-protein, wheat, barley rye, oatmeal, egg, hazelnut, peanut) is “hyper allergic” and explains that the home has to be “clinically clean”; even a bread crumb may trigger a reaction. He has his separate plates, cutlery and pans and knows that the other family members cannot sit “too close to him”. At school he has a buffet made just for him. Similarly, to other parents, this mother comments on how difficult it might be to be singled out because of the allergy. She tries to “turn it around a bit, well you get your food served, I mean that it can be a bit fun if everyone is envious because he gets his tray with food”. Another example is a father who realized his daughter was sad when the staff separated her from the other children during meal time, since she (2 years old, allergic to egg, milk-protein) had had a severe allergic reaction at nursery and had to go to hospital in an ambulance. He explains that the staff noticed that she was sad and therefore “moved her to sit close to the door, so she would sit closer to the others”. In line with his reasoning other parents argue that certain routines and allergy policies at school might influence the child's social life.

### The allergy condition as constantly present in the child's body

The analysis reveals that the allergy is understood as embodied; it is constantly present, “silent” and disguised in the child's body. It becomes visible as an allergic reaction when the child comes into contact with the allergen/s or via medical assessment. Food allergy risk is thus localized to the child's body, as something internal, existing all the time, depicted by some parents as a “ticking bomb”. For example, this mother describes the dramatic course of events when they discovered her daughter's allergy. The first time her daughter tasted a cashew nut her lips swelled and she began to cough. At the emergency department, her reactions suddenly became worse:

they started to treat her and then there were more and more spots, rashes all over, and I asked several times if this is ok, when will it stop, and she is suffering. And they say this is how it can be, no reason to worry, but then everything goes wrong because she starts to vomit, and excrete, and blood pressure goes down and red phones and people everywhere and we had to stay in the hospital (6 years old, allergic to cashew, hazelnut)

The mother explains that this experience was a “very traumatic 24 hours”. She felt that her daughter's allergy was a “matter of life and death”. Afterwards her allergy toward cashew was confirmed and they were prescribed an Anapen. The mother comments; “I felt I was bringing a ticking bomb with me when we left the clinic, later on it has become much better”. She uses a strong metaphor when she talks about her daughter as a “ticking bomb”. It implies that something could happen at any time and that the reaction may be powerful. Her account is a typical example that allergic reactions often emerge suddenly and unexpectedly, and that parents often seek hospital emergency care when the child has their first reaction.

Some parents discuss the idea that the food stuff could trigger a latent allergy and that an avoidance of the allergen is necessary in order for the child to outgrow the allergy. This mother whose 4-year-old son is allergic to peanut says that she “regrets that she gave him those nuts”. Another mother responds that she has also thought about that, but at some point in time her daughter, 4 years old, would have tasted nuts, and so they would have found out she is allergic. Her reasoning indicates that it is not possible to avoid the allergy by not exposing the child to the allergen. However, there is also an idea that by avoiding contact with the allergen, the allergic condition will not be “added to” and “expanded”:

I think this is difficult when they say you can eat a small amount of something, to provoke the body, this is what many people think, but many people also say that it will be like a ticking bomb, that the body will eventually explode. I have a friend who has a son who has always had tomatoes, until one day he had some ketchup, at the same time as hard physical exercise, and then it said “boom” and he had to go quickly to the emergency department. The doctor said you cannot be allergic to tomatoes, but obviously he was

This mother uses the metaphor “ticking bomb” to depict the ways food allergy can be added to if you expose the body to the allergen. At some point in time the body will “explode” in a powerful allergic reaction. This reasoning also implies that there are at least some possibilities to control the development of an allergy, as a parent of a food allergic child by not exposing the child to the allergen(s). A father refers to the “allergy doctors” he has met and argues that a prerequisite for an egg allergy to be outgrown is that the child does not consume eggs:

as the doctor said, if you manage to stay away from the eggs for some time, there is a good chance of the child outgrowing the allergy, so it is a matter of not adding to the allergy, try to keep him away from eggs, and when he reaches school age it will perhaps have disappeared

Parent's accounts further demonstrate how food and eating practices might become problematic for their children, but also impact on their own wellbeing, due to the stressful situation of not being able to feed their children properly or of the risk of transmitting allergens, for example, via breastfeeding. The mother recollects that her baby, now 6 years old, literally incorporated potential allergens through her own body via breastfeeding, an example of inter-embodiment, as the threat of illness comes from the body of the mother “into” the child:

the child health clinic advised me to test different things, to not have milk or eggs. But whatever I did, nothing became better, I lost weight because I didn't dare to eat anything, and finally, when I avoided everything, I demanded an allergy test even though she was only 5 months, and it turned out to be several things such as eggs and milk and I actually stopped breastfeeding because it was easier to know what she had when having formula

This mother's own body is affected as she has to avoid the potential allergens in her own diet, which has consequences when she loses weight during this period of “trial and error”, before the child's allergy was confirmed. Similarly, Brownlie and Leith (2011) in their UK study of MMR immunization, argue that parents' “sense of self is shaped through embodied interaction”, such as the piercing of the infant's surface and the anxieties related to that. They take their point of departure in an understanding of the infant's skin as a “site of *relationality*”(page 202); a place where self, others and societal imperatives intermingle. They emphasize that we need to understand the parental responsibility in this context of inter-corporeality. Here, empirical examples demonstrate that food consumption involves inter-embodiment aspects. Together with imperatives to eat healthy food and ideas about “good” parenting (Lee et al., 2010; Lupton, 2011) it is reasonable to assume that their difficulties of feeding their children shape parents' sense of self.

In the above examples, the risk of exposing the child to allergens is presented as two-fold; there is the more immediate risk of an allergic reaction, and the longer-term risk of building on or adding to the child's allergy. In line with Lupton (2011) and Brownlie and Leith's (2011) studies parents' give explanations and account for how they understand that the child's body may react to threats from outside. Lupton (2011) demonstrates how mothers discussed the importance of building up the infant's immune system by not exposing the baby to the risk of too much infection in this early lifestage. Here, drawing on their own experiences, together with medical information, alongside references to a “general” knowledge about causes of allergies, parents discuss how the allergy could be avoided or built on if the child is exposed to allergens. Parents do not explicitly mention the immune system but their suggestions are in line with biomedical knowledge; their accounts imply that the immune system does get sensitized to an allergen when exposed to it and then continues to react if triggered by that allergen^3^.

### Helping the child to identify, interpret and respond to bodily reactions

There are many variations in allergic reactions. Also, the seriousness of reactions might be difficult to judge. Crucially, in an allergic episode, it is the child's life that is at stake. It is against this backdrop that some parents emphasize that it is important that their children learn to recognize their own bodily reactions: “she recognizes her own symptoms, so she can say that now I feel this tickling on my tongue or now I will start to cough” (6 years old, allergic to egg, hazelnuts, cashew, pistachio). Some children have learnt to recognize when something is wrong:

he had the coconut soup, and felt it was perhaps not right and asked the staff, was there coconut in the soup, and yes, then he felt in his body that there was something, and then they started to act (went to see the school nurse) and this sort of recognizing in his body, I think that is so important, to recognize the symptoms because then you can stop things (14 years old, allergic to nuts, peanut, coconut, almond)

This mother focuses on her son's ability to “know through the body” and thus to recognize an allergic reaction. This also means, she underlines, that he can get help in case of a reaction. Another example is a mother who explains that her 15 year old son is able to discriminate between a mild and a severe reaction. “He knows the difference”; in his case a mild reaction means an itchy throat in contrast to a tingling effect in the body and paleness, which indicates it is a more severe reaction. This knowledge is so important since a reaction can evolve rapidly and might be life-threatening. She says that it “happens so quickly for her son”, who is allergic to almond, nuts and seeds. Within 10 minutes he has a reaction, and she has “the fear that he should die”, that you don't have enough time to do anything”. Another mother comments that in their case it is “the opposite”. It can take 3 hours before her daughter reacts, but then it can be “dramatic”. She says that now, at 10 years of age, her daughter has “learned to feel this (symptoms of allergy)”. This mother says that she hopes that in the future her daughter, allergic to milk-protein and nuts, will “dare to trust herself and not just others”. Parents express that with growing age children should be given opportunities to be more independent and learn from their experiences. But that they also experience conflicts in “letting their children go” and at the same time having to remind them of being constantly vigilant. A mother, whose daughter is 9 years old, addresses this contradiction, reflects about the future and says that she wants her daughter to be allowed to be “free and young”, but at the same time has to make her attentive to the risks associated with the allergy. Another mother says it is difficult for her when her son, 15 years old, goes away on his own, but that she really tries to not show her fears to avoid passing on her own anxiety. She feels he probably is a “bit worried about having the condition”. In line with Bruno de Sousa et al. (2022) study, with parents of children with chronic kidney disase, the results here demonstrate that the parenting of children with chronic conditions or lifelong diseases in many ways is challenging. It involves the parental responsibility to support children to be autonomous and at the same time give children “prolonged, constant and intensive—but unobtrusive—attention and support” (Bruno de Sousa et al., 2022, page 18). We argue that this imperative to consider somewhat contradictory approaches regarding the child's autonomy can be understood as the complexity to striving to *balance* the support within the parent-child-health/illness triad.

Throughout the material there are examples of how parents, regardless of the child's age, talk with their children about the food allergy, to help them understand and manage the risks. As has been demonstrated; children show resistance in different ways. Parents do not describe outright rebellion from their teenage children, but give examples of how their children on occasions “forget” to bring their adrenaline injector or do not “stand up for themselves” in every situation. One mother describes that her son, 15 years old, was “thin as a rake” after the summer camp, she believes that he didn't dare to eat the food served. These examples could be understood as a form of resistance toward parental control and/or that children are in the midst of a learning process to manage their food allergy. The above examples further demonstrate that some symptoms of allergy, such a tingling effect in the body, are invisible to others. The child can feel them and in order to get help, needs to make others aware of them. Other symptoms, such as a cough or paleness, can be observed by others, but still need to be identified as symptoms of food allergy to be responded to as such. This shows children's competence to recognize the feeling of becoming unwell is based on experience rather than age. Following Merleau-Ponty ((2002[1962])) this knowledge is entrenched in their embodied existence. These children are able to interpret their own body; they are *living* the experience of allergic reactions, feeling and naming the sensations. Thus, it is clear that children with food allergy take responsibility for their own health from a young age and act more independently with growing age. As highlighted by Bluebond-Langner (1978), Alderson (2007) and Brady (2014) lived experience provides children with relevant knowledge, leading them to competent decision-making, particularly regarding issues important to them. Yet, their parents also play an active role in the inter-embodied experience as they narrate their experiences. The results also demonstrate that with growing age the children will face new challenges when they are acting more independently in different arenas. Drawing on the sociology of childhood perspective this demonstrates the context-bound aspects of children as social actors (Holloway et al., 2019). To summarize, by utilizing the concept of inter-embodiment the results demonstrate, from the parental viewpoint, how their experiences of managing food allergy within the family intertwine with that of their children in a parent-child-health/illness triad.

## Discussion

Taking an inter-embodiment perspective on parental meaning-making of this case of child illness, food allergy, demonstrates that the character of the parental vigilance is not just a heightened awareness, but an active, constantly on-going process of closely observing and interpreting the child's body and behavior, and of helping the child to recognize bodily reactions of allergy. As we have seen through the deployment of qualitative methods which allow for in-depth exploration, the parents acquire an embodied, practical knowledge of how to manage the allergy. One advantage with focus group interviews as we have seen in this study is that the interaction between the participants and the dynamics in the focus group trigger reflections on the topics discussed and more elaborate accounts to make individual experiences understandable to other participants. Further, whilst we recognize that children themselves did not participate in this study the findings demonstrate important issues about children with food allergy as health actors. Adopting a childhood studies perspective enables an analysis of children's actions and voices within inter-generational relations; in this case, within the parent-child-health/illness triad. At the same time a limitation of the analysis is that we explore this triad from the parental perspective. Thus, aspects of the intersubjective relationships between children with food allergy and their parents might be overlooked, which data from children could have shed light on. There are also limitations in drawing on verbal accounts in exploring bodily issues. Thus, to further advance knowledge of the lived experience of child food allergy or other chronic conditions additional studies, preferably ethnographic approaches, are needed that explore the experiences of *both* parents and their children.

Parental anxiety and vigilance are not unique to parents of children with food allergy, but involve the management of other chronic conditions, such as Type 1 diabetes (Sullivan-Bolyai et al., 2003; Niedel et al., 2013; Rifshana et al., 2017), and hypoplastic left heart syndrome (Meakins et al., 2015). Here, Type 1 diabetes is especially relevant since there are similaries between the management of food allergy and Type 1 diabetes. Both food allergy and Type 1 diabetes are potentially life-threatening conditions that profoundly affect daily life. The individual has to control her eating to avoid allergic reactions or to maintain an even blood sugar level. Food allergy management also involes the administration of adrenaline in cases of severe reactions (Simons, 2010). In addition to diet management and to monitor the childs activity level, diabetes management also requires the need to regularly perform glucose control and to administer insulin (Doyle and Grey, 2010). Sullivan-Bolyai et al. (2003) and Rifshana et al. (2017) use the term “constant vigilance” to capture the circumstance that parental vigilance is a constantly ongoing process to manage the childs diabetes. This care-giving experience, the constantness and the never-ending endeavor to manage the child's condition has been explored by the first author and colleagues in an earlier study of parents of children with food allergy (Stjerna et al., 2014).

What seems to be an important characteristic of the parental vigilance in both cases is the threat of what *could* happen if not paying enough attention or not taking the right measures. Parents of children with food allergy depict food allergy as life threatening, a “death risk” lurking in the background, more or less constantly present in different everyday situations, amounting to an existential condition in parenting (Stjerna et al., 2017). Here, as we have seen, the empirical examples demonstrate parents experiences of their children having severe allergic reactions and being in need of intensive care, as well as less dramatic situations when they “read” their children's bodies to dectect potential signs of illness. Ultimately the child's life is at stake. This entails that *if* the child comes into contact with an allergen/s an ordinary situation might suddenly change and become dangerous. In a similar vein constant vigilance is strongly connected with the parents fear of hypoglycemia and the worry of not being vigilant enough to prevent the the long-term complications associated with Type 1 diabetes (Sullivan-Bolyai et al., 2003). Niedel et al. (2013) who have coined the term “parallel vigilance” to capture the process of how parents learn to discern symtomps of diabetes from other types of symptoms and reactions in the child, demonstrate how parents learn to manage the child's diabetes with more accuracy and confidence over time. The parental vigilance needed to manage children's chronic conditions is therefore to be understood as a certain form of lay expertice. Focusing on the more embodied aspects of this parental vigilance, we can detect similaries to the the care-giving experience of parents of children with Type 1 diabetes. Rifshana et al. (2017, p. 3231) found that parents of children with diabetes “spoke about the embodied experience of caregiving in terms of control and surveillance” but that their attempts to control were undermined by the unpredictable body of the child. Similar to the food allergy case, this unpredictability urged parents of children with diabetes to pay on-going attention to the childs body and actions, and to individualize their care-giving responses. But even with careful planning outcomes were not guaranteed (Rifshana et al., 2017).

Here, the inter-embodiment lens on the parental perspective on the *relations* between children and parents adds to this understanding and reveals how *bodily* interconnected and intertwined parents' and children's lives are. We argue that inter-embodiment is a concept that bridges relations between parents and children as social actors, (…) “children and adults negotiate the status of the child's body and emotions, in the daily give-and-take of relational processes” (Mayall, 2015 p. 313). As Lupton (2012, p. 40) puts it “each body's ‘being-in-the-world' is shaped by the other's”. What comes through in this study is that children with food allergies could be characterized as being in a state of liminality; a position *in between* different categorizations, such as *neither* healthy or ill (see also Stjerna, 2018). This means that mostly the child is symptom free but at the same time constantly exposed to allergens and the *risk* of becoming ill. The notion of liminality has been used to explore how adults with chronic conditions experience liminal spaces in between health and illness in their everyday life (see eg. Jackson, 2005). But here the analysis demonstrates that parental vigilance is the embodied manifestation of concern for children's bodies which is in perpetual liminality. As children can go from being “healthy” (asymptomatic) to very sick in a moment, parents need to be constantly vigilant. Parents use all of their senses to identify signs of illness in their children and are attentive to their children's bodily expressions as part of their vigilance.

This study further demonstrates that the agency of children with food allergy and parents is intertwined in a complex web of *social* relations, including a range of actors, such as health care personnel, dietitians, and pedagogues at day care and school. This is line with a study of Swedish and Scottish parents' that depicted that different places where the child with food allergy spent time not only demanded different types of risk management but also could vary within the same space from day to day and was depending on several actors (Stjerna et al., 2017). Here, the interactive perspective on inter-embodiment adds to this complexity. It demonstrates how *material* circumstances, such as allergens hidden in food, how the body reacts to an allergen/s and medical treatment to stop an on-going reaction, influence allergy management. Throughout, the analysis of this material clearly demonstrates parental conceptualizations of the child's allergic body as both external and internal. Ideas about the the “external body” are manifested in the ways the parents “read” the child's body to identify potential illness, using their own senses, across minor to serious cases of food allergy. The empirical examples demonstrate that the ubiquity of allergens in many situations, together with the potential severity of allergic reactions, are material circumstances that shape the agency of the child and their surroundings. The health precautionary measures even affect closeness between the child and others, which entails that children may face social risks in situations involving food. Ideas about the “internal body” surface in the parents' understandings of the condition as constantly present in the child's body, depicted by some parents as a “ticking bomb”. The metaphor “ticking bomb” implies that allergic reactions might be severe and even life-threatening, arise unexpectedly and, despite precautions, are difficult to avoid altogether. So, despite that parents and children learn to recognize symptoms of allergy over time and thus can exercise some control over the allergy, parents' accounts also demonstrate that the allergy entails this threatening aspect of sudden and severe reaction. In addition, the unpredictability of reactions requires constant vigilance and individualized responses. In addition to parents' understandings of their children's immediate reactions they also discuss the more long-term implications of food allergy avoidance or exposure, in the context of medical advice. The uncertainty of competing discourses of food allergy (different hypotheses of what causes food allergy) and what is the right thing to do, adds to complexity and parental responsibility, which has also been demonstrated in the case of MMR immunization (Brownlie and Leith, 2011).

We argue that parental vigilance has to be understood in the context of inter-corporeality where societal imperatives intermingle with the parental embodied interaction and experience of managing child food allergy. To eat properly is key to maintain health; in Olin Lauritzen's (1997) study the quality of the baby's feeding was used by mothers' as a basic indicator of the child's health, and if perceived as not sufficiently good, subject to mothers' actions. However, this very fundamental imperative to properly feed the child, is actually challenged in cases of food allergy. Here, parents accounts address these issues when they for example narrate about their children's reluctance to eat after having severe reactions. In the context of moral parenthood it is reasonable to assume that such difficulties not just are challenging in the everday practical management of the child's allergy, but also have the potentiality to affect parents sense of themselves as “good parents”(see eg. Lee et al., 2010). The intertwinedness of parents and childrens lives further entails that parents bodies and wellbeing are affected and that bodily boundaries sometimes get blurred, such as in times of breastfeeding. In those cases it is not just the child's food allergic body that is at risk. Also, the mother's own body might get affected by a restricted diet. Parents also express that the constant vigilance means that they experience the emotions of anxiety and worries and try not to pass their own anxiety to their children. This also highlights how the more emotional boundaries between parents and children might get blurred when managing the child's allergy.

In this context of inter-corporeality the results of Henderson et al. (2020) are relevant. They adopted the analytical lens of inter-embodiment in a a study with families who were members of the Choctaw Nation of Oklahoma in the United States who had a child with Type 1 diabetes. These parents who, due to tribal membership had relatively good access to medical care, still experienced anxiety because of the daily glycemic control of their child's diabetes. The results demonstrate how potentially fatal outcomes required parental hypervigilance. This constant vigilance and lived experiences of their children's disease resulted in parents experiencing a stress-induced toxic condition, a kind of embodiment Henderson and colleagues term “diabetes-by-proxy”. This psychological embodiment of their childrens disease reveals ‘the parent as such a close disease-partner with the child that they experience everything about the condition minus only the physical sensations concurrent with it' (page 35). Thus, this kind of embodiment is virtual as parents have no symptoms of Type 1 diabetes, but cognitively engulfe their children's metabolic physiology, behaviors and mood. Henderson et al. argue that the parents' absorption of the child's Type 1 diabetes is more than an intellectual endeavor to mangage their childrens condition. It is a way to take virtual control of the death threat that is ever present in the child's body. In a similar vein our analysis reveals that parents of food allergic children are heavily influenced by the uncertainty of the condition and the potentially life-threatening outcome of allergic reactions, which urge parents to be constantly vigilant. The results also demonstrate how the embodiment aspect of this vigilance entails the interconnected food allergy experience of parents and children.

Further, dependency on others is an underlying theme throughout the material presented here and what comes through is the *relational embodied agency* of parents and children. Importantly, childrens' agency is inflicted by power relations. Adopting the inter-embodiment lens these childrens' resistance is associated to the lived experience of allergic reactions and medical treatment and the stress and fear such experiences arouse. Children show reluctance to eat after having a severe reaction or try to resist intensive medical care. Thus, children are far from passive bodies; they recognize symptoms, they make others aware of their sensations and in some instances try to avoid food or medical treatment, evidence of powerful bodily responses to their lived experience. Parents accounts also show how the interaction with service providers, such as health care personnel and dietitians, may be of special importance in some situations in supporting parents to manage those situations. In developing our thinking about children/parents/health and illness it is evident from the above illness narratives that the identification and response to risk (of allergic reaction) is shared, taking place in social interaction between the child, their parents and sometimes health care professionals. Whilst it may be difficult to articulate what takes place in this interaction we contend that the concept of inter-embodiment comes close to explaining the complexity of children's competency in managing their own bodies as they develop experiential knowledge intertwined in embodied relation to parents.

The role of parents is to prevent danger and mitigate risk whilst not being over-protective, which is regarded as a risk in itself. “Wrapping children in cotton-wool” is seen as highly undesirable (Jenkins, 2006). But here we have demonstrated how parents are learning to trust that their child knows their own body and encourage their children to give voice to their experiences and help them to interpret their sensations and feelings. Parents accounts also demonstrate the complexity of this endeavor since children with food allergy in many ways are more “at risk” than other children. They describe tensions in “letting their children go” and at the same time making their children aware of the risks, which have also been demonstrated in the case of chronic kidney disease (Bruno de Sousa et al., 2022). Adding to this, earlier research demonstrates that risk and trust can be seen as closely related in the management of food allergy and that parents “on the one hand have to encourage their children to be constantly aware of food allergy risk and on the other hand develop trust in their children as risk managers and let them live as normal lives as possible” (Stjerna et al., 2017, p. 364). Taken together, these results challenge the simplistic view and dominant discourse which characterizes parents as being over-protective. Thus, our analysis supports McLaughlin and Goodley (2008, p. 323) argument that there is a need to inform social theory by exploring “the day-to-day contingent and situated agency that people enact” when living with disabled, and we argue, chronically ill children. Children with complex health issues or long term health conditions, alongside children with disabilities, are often regarded as being more vulnerable and at risk of various forms of abuse. Parental actions can be misinterpreted or misunderstood by health and social care professionals, perceiving vigilance to be over-protection or intensive behavior that denies children any autonomy. Such perceptions, including carer anxiety, are often a focus of child in need or child protection concerns and assessments. Yet most often parents and carers of children with long term conditions, such as food allergy, are following medically recommended regimes and trying to comply, however they also know their own child and when recommended routes may not be appropriate. Increasing the capacity of parents and carers to care and safeguard their children involves professionals recognizing that parents are a source of support and information and can be relied upon to interpret and to explain signs, symptoms, behaviors of children, their knowledge should be valued by professionals who interact with the child and their family. Ultimately, the needs of the child should remain at the forefront and children should be consulted in matters affecting them, to gradually decrease their dependence on parental carers and to increase their independence. According to Mayall (2015) parents can contribute to children's health and wellbeing in ways other than health, welfare and education service providers often recognize, stemming from their lived experience with their children. Without this recognition parental views and lay-perspectives more generally risk being undervalued. To accomplish successful management of the child's chronic condition, valuing the parental perspective within healthcare interactions is pivotal. The adoption of this perspective of inter-embodiment uncovers a form of embodied knowledge that is often not verbalized and can serve to enhance understandings of the parental perspective of managing child food allergy. These findings offer a new perspective to health and social care professionals who aim to enhance health care and social support for families who experience the complexity of managing food allergy or other chronic conditions, in the daily life of their child.

## Conclusion

Taking our point of departure in the parent-child-health/illness triad to explore the embodied aspects of parental vigilance in parenting children who have a food allergy, utilizing the concept of inter-embodiment, has proved to be useful. Acknowledging the limitation of focusing solely upon the parent perspective, this conceptual lens recognizes the interconnected food allergy experience of parents and children. The parents acquire an embodied, practical knowledge of how to manage the allergy. This inter-embodied parental vigilance is constantly carried out trying to exercise some control over the allergy, but the unpredictability of sudden and severe reactions remains. The allergy management also entails that parents are learning to trust that their child knows their own body. They help their children to recogonize and interpret symtoms of allergy and encourage them to voice their experiences. However, the character of the potential food allergy risk, the life and death aspect, makes this endeavor especially challenging and accentuates the existential dimension of this care-giving experience. Further, parents experience the constant vigilance as an inter-embodied experience in terms of their own wellbeing and bodies might be affected by managing their children's food allergy. Thus, adopting an inter-embodied lens focusing on the parental experience of managing chronic child illness demonstrates how the illness experiences, in different ways, transcend individual bodies.

## Data availability statement

The raw data supporting the conclusions of this article will be made available by the authors, without undue reservation.

## Ethics statement

The study was approved by the Ethics Committee at the Karolinska Institutet (Nr: 2008/569-31, 2012/1051/32). The patients/participants provided their written informed consent to participate in this study.

## Author contributions

M-LS has initiated and formulated the conception and design of the article, collected the data, and together with GB carried out the analysis of the material. All authors contributed to the development of the theoretical perspectives, concepts utilized in the qualitative analysis, manuscript revision, read, and approved the submitted version.

## Footnotes

^1^The project ‘*Dangerous food' and child health: Living with food and eating risks of everyday life. A qualitative study of parents of food allergic children'*, was headed by professor Sonja Olin Lauritzen, Department of Education, Stockholm University and carried out by the first author (M-LS), and Med Dr. Mirja Vetander, as part of a larger research program headed by Professor Magnus Wickman and associate Professor Gunnar Lilja ‘*Severe child food allergy: from diagnostic to practice*' at the Sachs Children's and Youth Hospital in Stockholm. For full details of the study see Stjerna et al. (2014).

^2^All namnes are fictious.

^3^Food allergic reactions occur when the body's immune system erroneously targets a non-harmful food as dangerous. This sets in motion an immune response that in most cases causes a reaction mediated through the antibody immunoglobulin E (IgE). IgE attach to mast cells and basophils, which leads to an immediate release of histamine and cytokines causing the symptoms of the reaction.
